# Household air pollution and COPD: cause and effect or confounding by other aspects of poverty?

**DOI:** 10.5588/ijtld.21.0570

**Published:** 2022-03-01

**Authors:** K. Mortimer, M. Montes de Oca, S. Salvi, K. Balakrishnan, R. M. Hadfield, A. Ramirez-Venegas, D. M. G. Halpin, B. Ozoh Obianuju, K. Han MeiLan, R. Perez Padilla, B. Kirenga, J. R. Balmes

**Affiliations:** 1 University of Cambridge, Cambridge, UK; 2 Liverpool University Hospitals NHS Foundation Trust, Liverpool, UK; 3 Hospital Universitario de Caracas Universidad Central de Venezuela and Centro Médico de Caracas, Caracas, Venezuela; 4 Pulmocare Research and Education (PURE) Foundation, Pune, India; 5 Sri Ramachandra University, Chennai, India; 6 Australian Institute for Health Innovation, Macquarie University, Sydney, NSW, Australia; 7 Departamento de Investigación en Tabaquismo y EPOC, Instituto Nacional de Enfermedades Respiratorias, Mexico; 8 College of Medicine and Health, University of Exeter Medical School, University of Exeter, Exeter, UK; 9 Department of Medicine, College of Medicine, University of Lagos, Lagos, Nigeria; 10 University of Michigan, Ann Arbor, MI, USA; 11 Makerere University Lung Institute, Kampala, Uganda; 12 Department of Medicine, University of California, San Francisco and Division of Environmental Health Sciences, School of Public Health, University of California, Berkeley, CA, USA

**Keywords:** household air pollution, COPD, lung disease, LMICs

## Abstract

**SETTING:**

Household air pollution (HAP) and chronic obstructive pulmonary disease (COPD) are both major public health problems, reported to cause around 4 million and 3 million deaths every year, respectively. The great majority of these deaths, as well as the burden of disease during life is felt by people in low- and middle-income countries (LMICs).

**OBJECTIVE AND DESIGN:**

The extent to which HAP causes COPD is controversial; we therefore undertook this review to offer a viewpoint on this from the Global Initiative for COPD (GOLD).

**RESULTS:**

We find that while COPD is well-defined in many studies on COPD and HAP, there are major limitations to the definition and measurement of HAP. It is thus difficult to disentangle HAP from other features of poverty that are themselves associated with COPD. We identify other limitations to primary research studies, including the use of cross-sectional designs that limit causal inference.

**CONCLUSION:**

There is substantial preventable morbidity and mortality associated with HAP, COPD and poverty, separately and together. Although it may not be possible to define clear causal links between HAP and COPD, there is a clear urgency to reduce the avoidable burden of disease these inflict on the world’s poor.

## Abstract (fr)

**CONTEXTE:**

La pollution de l’air intérieur (HAP) et la bronchopneumopathie chronique obstructive (COPD) sont des problèmes majeurs de santé publique, respectivement à l’origine d’environ 4 millions et 3 millions de décès chaque année. La grande majorité de ces décès et le poids sanitaire de ces complications durant la vie touchent les personnes vivant dans les pays à faible revenu et revenu intermédiaire (LMIC).

**OBJECTIF et MÉTHODE:**

L’ampleur de la responsabilité de la HAP dans la COPD est controversé. Nous avons donc réalisé cette revue pour présenter le point de vue du Global Initiative for COPD (GOLD).

**RÉSULTATS:**

Bien que la COPD soit bien définie dans de nombreuses études sur la COPD et la HAP, des limites considérables en matière de définition et de mesure de la HAP ont été observées. Il est donc difficile de dissocier la HAP d’autres caractéristiques de la pauvreté, qui sont elles-mêmes associées à la COPD. Les études de recherche primaire présentaient d’autres limites, dont l’utilisation de méthodes transversales qui limitent l’inférence causale.

**CONCLUSION:**

Une part conséquente de la morbidité et de la mortalité associées à la HAP, la COPD et la pauvreté peut être évitée, que ces dernières soient considérées séparément ou ensemble. Bien qu’il ne soit pas nécessairement possible de définir des liens de causalité clairs entre la HAP et la COPD, il est urgent de réduire le poids sanitaire évitable de ces maladies sur les pays pauvres.

The WHO estimates that roughly 4 million people die prematurely every year as a result of exposure to household air pollution (HAP). Of these deaths, around one third (1.5 million) are attributable to chronic obstructive pulmonary disease (COPD). The more recent Global Burden of Disease (GBD) study estimates suggest that the number of deaths related to HAP is lower than this, but nevertheless substantial, at 2.31 million deaths,^1^ which accounts for 3.6% disability-adjusted life-years (DALYs).^2^,^3^ COPD itself is a leading cause of death globally – the third most common cause of death – responsible for just over 3 million deaths a year.^4^

HAP is caused by the use of dirty-burning fuels to provide energy for cooking, heating and lighting. Almost all the people living with and dying from HAP-associated COPD are born into households in low- and middle-income countries (LMICs), by which time their exposures to COPD risk factors have already started.^5^

This paper aims to highlight HAP and COPD as two major causes of avoidable global morbidity and mortality and to explore the nature of their relationship. We do this by posing four questions and suggesting priorities for action now and priorities for future research: 1) How is HAP measured in studies on HAP and COPD? 2) How is COPD diagnosed in studies on HAP and COPD? 3) Are the respiratory symptoms and spirometric abnormalities commonly observed in association with HAP exposure in LMICs due to COPD? 4) Is the phenotype of fixed airflow obstruction associated with HAP similar or different from tobacco smoke-induced COPD?

## HOW IS HAP MEASURED IN STUDIES ON HAP AND COPD?

Although conceptually the definition of HAP is relatively straightforward, its meaningful measurement has been challenging.^6^ One of the big challenges is that HAP exposures can start in early life (from conception or before) and continue – but vary – throughout life. There has thus been much reliance on the use of questionnaires to try to get a picture of life-cycle exposures, but these are rather blunt tools and are limited by recall bias. The development of a robustly validated estimate equivalent to cigarette smoking pack-years would be of great value; however, this has been proven elusive and is likely to remain so, given the greater difficulty of quantifying HAP exposure vs. the number of cigarettes smoked. No equivalent cooking quantity exists that is comparable to the relatively standard quantity of one cigarette. HAP exposure is often defined as ‘exposed’ vs. ‘not exposed’, but this ignores complexities of concentration, duration of exposure within a 24-h period, constituents, indoor vs. outdoor environments, age at which exposure starts (including prenatal), duration of cumulative exposure (e.g., cooking-years). Some of these complexities can be addressed by direct measurement of HAP exposure, for e.g., by measuring particulate matter (PM), carbon monoxide (CO), black carbon (BC), polycyclic aromatic hydrocarbons (PAHs), alveolar macrophage carbon loading. However, these involve new limitations including providing only a snapshot of a lifetime exposure, technical complexities and cost. PM_2.5_ (particles with a diameter of ≤2.5 microns) and CO are the two pollutants that have been most commonly measured in HAP studies on respiratory health. Given the relatively large number of studies of exposure to ambient PM_2.5_ and respiratory health in high-income countries, PM_2.5_ has been the main focus of HAP research. Early HAP exposure studies showed 24-h indoor concentrations of PM_10_ (particles with a diameter of ≤10 microns) generated from solid fuels in different settings to be in the range of 300–3,000 μg/m^3^, with peaks reaching as high as 20,000 μg/m^3^ during cooking.^7^–^9^ In a study from Guatemala, the 24-h average PM_2.5_ generated by domestic wood burning was found to be 520 μg/m^3^,^10^ which is similar to findings from other studies from Nepal (680 μg/m^3^)^11^ and China (489 μg/m^3^).^12^ These reported concentrations are an order of magnitude higher than the WHO global ambient air quality guidelines.

Most of the early studies linking exposure to HAP and COPD did not use measurements of pollutant levels but relied instead on self-report of cooking with solid fuels or not. One of the first studies to measure HAP in relation to measurement of airflow obstruction was conducted in Mexico.^8^ In this study, peak 1-h indoor concentration of PM_10_ often exceeded 2 mg/m^3^. Another early study that measured both exposure to HAP and lung function was from China and found mean kitchen concentrations of PM_10_ in homes cooking with biomass fuels to be ~1 mg/m^3^; these measurements were made with a continuous reading instrument over 5-min periods.^13^ One of the first studies to measure personal exposure as opposed to area concentrations of HAP was from the RESPIRE randomised controlled trial of a chimney cookstove intervention. In this study, exhaled CO (eCO) was measured repeatedly and an association between eCO and decline in forced expiratory volume in 1 sec (FEV_1_) over an 18-month period was observed.^14^ A recent study in Malawi measured personal exposure to both PM_2.5_ and CO over a 48-h period at enrolment and annually over a 2-year follow-up.^15^ In this study, the median PM_2.5_ (77 μg/m^3^) and CO (1.27 ppm) were not associated with a decline in FEV_1_, although a caveat is that cooking is commonly done outdoors in Malawi, except when it rains. While personal monitoring is generally preferred over area monitoring, all of the studies mentioned here measured HAP for brief periods so only a “snapshot” assessment of exposure was obtained. Given that cumulative exposures to HAP that may contribute to the eventual development of COPD begin in childhood,^16^–^18^ and perhaps even in utero or pre-conception, single or even multiple snapshot measurements of exposure in adulthood are likely insufficient.

Whether measured directly or indirectly, all measures of HAP are limited by the problem of confounding by other dimensions of poverty. It is therefore difficult to isolate the effects of HAP. This is well illustrated by the energy ladder, where the most dirty-burning (animal dung and crop residues) and clean (gas and electricity) fuels are at opposite ends of the ladder, both from HAP exposure and cost perspectives. Arguably, HAP is itself a dimension of poverty – air quality poverty – and therefore essentially impossible to disentangle from other dimensions of poverty outside of artificially created study conditions (e.g., laboratory studies, intervention trials). Many of these other dimensions of poverty (e.g., poor early life nutrition, limited access to healthcare, tobacco smoke and other noxious inhalational exposures) are themselves known to be associated with the development and progression of COPD. We are therefore left with uncertainty around the extent to which any measure of HAP is an indicator of the direct harms of this exposure vs. harms related to its association with other adverse health effects of poverty. There is, however, no doubt that HAP is a very real problem, with high and unhealthy exposures to PM_2.5_ the daily norm for many people living in low-resource settings.

## HOW IS COPD DIAGNOSED IN STUDIES OF HAP AND COPD?

In contrast to the challenges of measuring exposure to HAP, there is a straightforward definition of COPD based on the results of post-bronchodilator spirometry. GOLD defines COPD as a “common, preventable and treatable disease that is characterized by persistent respiratory symptoms and airflow limitation that is due to airway and/or alveolar abnormalities usually caused by significant exposure to noxious particles or gases”.^4^ In many LMIC households, levels of HAP certainly qualify as such an exposure. Domestic combustion of biomass fuels generates smoke that is not dissimilar to tobacco smoke – a mixture of irritant gases and particles with complex hydrocarbons (e.g., polycyclic aromatic hydrocarbons) on their surfaces – without the nicotine.

Many studies of HAP and COPD have used robust definitions of COPD with high standards of quality-assured spirometry, although access to this for clinical practice/diagnosis is often limited in LMICs. Where airflow obstruction is found in association with HAP exposure in referral centres, it has often been found to be relatively milder than that typically observed in heavy smokers and typically without emphysema on computed tomography imaging. However, many people with heavy HAP exposure have chronic respiratory symptoms consistent with chronic bronchitis.^19^ A study of airway histology in non-smoking women with exposure to biomass smoke found airway remodelling with fibrosis without much emphysema.^20^ The problem of chronic respiratory symptoms/disease associated with HAP is therefore wider than spirometry-defined airflow obstruction. There is increasing evidence that small airway disease occurs mainly in non-smoking women. The type of inhalation that occurs during cooking activity is likely to lower the risk of damage spreading beyond the small airways, thus leading to an airway-predominant COPD phenotype.^21^ Recent evidence suggests that small airway disease is the predominant phenotype in non-smoking women, and that oscillometry may be a useful diagnostic test.^22^

## ARE THE RESPIRATORY SYMPTOMS AND SPIROMETRIC ABNORMALITIES COMMONLY OBSERVED IN RELATION TO HAP EXPOSURE IN LMICS DUE TO COPD?

Although a considerable burden of disease has been attributed to COPD due to HAP exposure, this is somewhat controversial as there are conflicting data and opinions.^23^–^28^ Given that there have been several recent systematic reviews and meta-analyses examining this association, we brought these together (see Box 1, Supplementary Data, for search strategy) to help answer this question (Table 1). These reviews draw on a similar pool of studies and data, but differ in methodology (e.g., criteria for study inclusion, definitions of exposure/outcome, etc.). These are nevertheless consistent in finding a positive association between HAP and COPD. They are also consistent in being affected by the limitations in the definition of HAP exposure (questionnaire-based) and outcome assessment (often without spirometry or spirometry without bronchodilator administration), as discussed above. Therefore, these reviews leave uncertainty as to the extent to which the association can be explained mechanistically by exposure to HAP vs. other dimensions of poverty. In addition, most of the studies included in the systematic reviews and meta-analyses are cross-sectional which limits causal inferences, and are subject to several other limitations.

**Table 1:** Summary of systematic reviews and meta-analyses of studies of COPD and household air pollution since 2015

Study	Year	SR/MA	Inclusion criteria	Studies included *n*	COPD and HAP definitions	Key conclusions	COPD-specific conclusions
Saleh^45^	2020	SR	RCTs on the clinical effectiveness of interventions to reduce particulate matter in LMICs	14 included studies, 12 tested ’improved’ cookstoves, most using biomass	Clinical diagnosis of COPD Any household-level intervention to reduce exposure to air pollution, as determined by particulate matter exposure of any size classification	Evidence from the RCTs performed to date suggests that individual household-level interventions for air pollution exposure reduction have limited benefits for respiratory health	Only one COPD specific study included 4-year integrated COPD management/prevention intervention, which was associated with spirometric improvements in the intervention group
Pathak^49^	2020	SR/MA	Case-control, retrospective cohort, cross-sectional studies and conducted in adults that assessed COPD using any diagnostic criteria	A total of 35 studies with 73,122 participants were included	COPD based on standard diagnostic criteria Indoor air pollution due to biomass cooking fuel	The pooled analysis showed that exposure to indoor air pollution due to solid biomass fuels increased risk of COPD by 2.65 (95% CI 2.13–3.31; *n* = 73,122) and CB by 2.89 (95% CI 2.18– 3.82) times more compared to non-biomass fuels	The risk of COPD was higher in Africa region (OR 3.19), Asia (OR 2.88), South America (OR 2.15), Europe (OR 2.30) and North America (OR 2.14)
Lee^3^	2020	SR/MA	Studies evaluating risk of adverse cardiorespiratory, paediatric, and maternal outcomes from exposure to household air pollution, compared with no exposure	476 studies (15.5 million participants) from 123 nations (99 [80%] of which were classified as low-income and middle- income) met the inclusion criteria	Studies evaluating the association between exposure to household air pollution and adverse cardiorespiratory, paediatric health outcomes Household air pollution exposure was defined as use of polluting fuels (coal, wood, charcoal, agricultural wastes, animal dung, or kerosene) for household cooking or heating	Household air pollution was positively associated with asthma (RR 1.23, 95% CI 1.11–1.36), acute respiratory infection in both adults (1.53, 95% CI 1.22– 1.93) and children (1.39, 95% CI 1.29–1.49), COPD (1.70, 95% CI 1.47–1.97), lung cancer (1.69, 95% CI 1.44–1.98) and TB (1.26, 95% CI 1.08–1.48); cerebrovascular disease (1.09, 95% CI 1.04–1.14) and ischaemic heart disease (1.10, 95% CI 1.09–1.11); and low birthweight (1.36, 95% CI 1.19– 1.55) and stillbirth (1.22, 95% CI 1.06–1.41); as well as with under-5 (1.25, 95% CI 1.18– 1.33), respiratory (1.19, 95% CI 1.18–1.20), and cardiovascular (1.07, 95% CI 1.04–1.11) mortality	Household air pollution associated with COPD (RR 1.70, 95% CI 1.47–1.97)
Sutradhar^50^	2019	SR	Studies that reported the prevalence and/or risk factors of COPD among Bangladeshi people	9 studies	Studies that reported prevalence of COPD or emphysema or CB or obstructive lung disease or single or multiple risk factors of COPD or emphysema or CB or obstructive lung disease Any risk factors were included (not HAP-specific)	Pooled COPD prevalence among Bangladeshi adult was 12.5% (95% CI, 10.9–14.1) using GOLD criteria and 11.9% (95% CI, 11.4–13.6)	Noted that there is a strong association between tobacco use and COPD; COPD prevalence higher among biomass fuel users (16.4–17.3%) compared to clean fuel (e.g., LPG or natural gas) users (4.4–10.0%); a high- quality study that collected data from nearly 4,000 urban and rural participants also found that biomass fuel users were six times more likely to have COPD compared to clean fuel users (OR 5.9, 95% CI 1.0-34.5; *P* = 0.047). Two moderate-quality studies found that biomass fuel use was positively associated with a higher prevalence of COPD among rural women*)*
Bellou^51^	2019	SR/MA	Umbrella review of systematic reviews and meta-analyses of observational studies on environmental factors and biomarkers for risk of COPD	19 eligible articles (8 systematic reviews, including 11 articles with 18 unique meta- analyses)	Studies examining the association of environmental factors or serum biomarkers and risk for COPD Environmental factors (not HAP- specific)	One included systematic review included 15 data sets on COPD and biomass fuels and reported OR 2.37 (95% CI 1.72–3.26) but small study/excess significance bias but highly suggestive of effect	About 30% of COPD patients are never smokers, indicating that there are additional factors modifying the risk for COPD; our umbrella review indicated that exposure to biomass fuels was also associated with the risk of developing COPD
Zhu^52^	2018	SR	Systematic review of articles on disease burden of COPD in mainland China in both Chinese and English published before October 2015	7 studies reported association of biomass fuel/solid fuel usage and COPD	Studies that considered the disease burden or quality of life of COPD	Need to refer back to original studies^11^,^27^,^37^,^45^,^48^,^52^,^53^ no further data provided	Tobacco exposure and biomass fuel/solid fuel usage were documented as two important risk factors of COPD
Thakur^53^	2018	SR/MA	(Quasi-) experimental studies of improved biomass cookstoves and outcomes (LBW), pre- term birth, perinatal mortality, paediatric acute respiratory infections and COPD in women (and their children) in LMICs	24 studies met eligibility criteria	Any risk factors (not HAP-specific)	Improved cookstoves provide respiratory and ocular symptom reduction and may reduce COPD risk among women, but had no demonstrable child health impact	No (quasi-) experimental studies assessing COPD among women were identified. In a pre-specified sensitivity analysis, improved cookstoves were associated with a 26% reduction in the incidence of COPD among women In observational studies, improved cookstoves were associated with a significant reduction in COPD among women: two studies (9,757 participants; RR 0.74, 95% CI 0.61–0.90)^29^,^30^ (both conducted in China)
Sana^54^	2018	SR/MA	Case-control or cross- sectional studies involving exposure to indoor biomass smoke	24 studies (5 case-control studies and 19 cross- sectional studies)	COPD as an airflow limitation that is not fully reversible (assessed by post-BD spirometry), either according to the American Thoracic Society/European Respiratory Society criteria^15^ (post- BD (FEV_1_/FVC) ratio ≤ 0.70) or the Global Initiative for Obstructive Lung Disease criteria^16^ (presence of a post-BD FEV_1_ the method of FEV_1_/FVC ratio <0.70) or FVC below the lower limit of normal value Women, cooking or cooking and heating with biomass fuels represented the exposure (intervention), cooking or cooking and heating with non-biomass fuel or clean fuel were the comparator	Biomass-exposed individuals were 1.38 times more likely to be diagnosed with COPD than non- exposed (OR 1.38, 95% CI 1.28–1.57) Spirometry-diagnosed COPD studies failed to show a significant association (OR 1.20, 95% CI 0.99–1.40); however, the summary estimate of OR for CB was significant (OR 2.11, 95% CI 1.70–2.52)	The pooled OR for cross-sectional studies and case-control studies were respectively 1.82 (95% CI 1.54–2.10) and 1.05 (95% CI 0.81–1.30) Significant association was found between COPD and biomass smoke exposure for women living in rural as well as in urban areas
Yang^55^	2017	SR/MA	Case-control or cohort design studies conducted in Mainland China assessing risk factors related to COPD	19 studies (8 of which reported on risk of biomass burning)	“Chronic Obstructive Pulmonary Disease” or “COPD” in combination with “risk factors” Not HAP-specific	Pooled OR for biomass burning risk of COPD was 2.218 (95% CI 1.308–3.762; *P* = 0.003)14,17,19,20,22,23,27,30	
Liu^56^	2016	SR	Any study design; mostly cross-sectional, case- control or cohort studies	107 studies included (21 of indoor pollution and COPD in LMICs	Search terms included “COPD exacerbation”, “air pollution”, “air quality guidelines”, “air quality standards”, “COPD morbidity and mortality”, “chronic bronchitis”, and “air pollution control” separately and in combination	The significantly increased OR for biomass cooking ranged from 1.86 (95% CI 1.16–2.99) in Brazil 8.7– 95.9) in Turkey 0.4–4.2) in India to 28.7 (95% CI for CB, 1.2 (95% CI– 15.0 (95% CI 5.6–40.0) in Mexico COPD, 9.7 (95% CI 3.7–27.0) overall to 75 (95% CI 18–306) when cooking was 200 hour-years in Mexico bronchitis and COPD combined, and 2.3 (95% CI 1.2– 4.4) to 2.9 (95% CI 1.7–5.1) for various respiratory symptoms	Biomass cooking in low-income countries was clearly associated with COPD morbidity in adult non-smoking females
Po^57^	2011	SR/MA	Studies on use of biomass fuels with respiratory outcomes in rural women and children	51 studies were selected for data extraction and 25 studies were suitable for meta-analysis	Respiratory-related disease, symptoms and functioning Exposure to biomass (wood, animal dung, crop residue, charcoal)	Another six studies measured COPD in women exposed to biomass fuels and women exposed to LPG, gasoline or oil;^29^,^33^,^36^,^39^,^40^,^46^ women exposed to biomass fuel smoke were 2.4 time more likely to develop COPD (OR 2.40, 95% CI 1.47–3.93); the random-effect model was used to adjust for heterogeneity among studies (*I*^2^ = 67.2%, *P* < 0.001)	Of the six articles that focused on COPD in women, two articles reported that <25% of the women in the community smoked, which was consistent with previous studies on smoking prevalence that is, that across different countries in several continents, in general, women in rural populations rarely smoke tobacco

COPD=chronic obstructive pulmonary disease; SR/MA=systematic reviews/meta-analyses; RCT=randomised controlled trial; LMIC=low- and middle-income country; CI=confidence interval; OR=odds ratio; RR=risk ratio; CB=chronic bronchitis; LPG = liquefied petroleum gas; LBW = low birth weight; HAP = household air pollution; BD = bronchodilator; FEV_1_ = forced expiratory volume in 1 sec; FVC = forced vital capacity.

Two large studies from China that assessed the impact of cookstove, cleaner fuel and/or ventilation interventions support a HAP-COPD association.^29^,^30^ Evidence of reduced incidence of COPD was observed in a retrospective cohort study after a major campaign to provide chimney stoves in a rural area of China.^29^ In a longitudinal study over 9 years, two interventions – biogas and kitchen ventilation – were compared to cooking as usual.^30^ Use of biogas and improved ventilation were associated with a reduced decline in FEV_1_ and risk of COPD; use of both interventions had greater effect than either alone.^30^

To examine the association of exposure to HAP and COPD further, and in particular, to determine how to assess exposure with a possible mechanistic association with COPD, we searched for original research papers on HAP and COPD published in the last 5 years (see Box 2, Supplementary Data, for search strategy) that assessed both post-bronchodilator airflow obstruction and quantified exposure to PM_2.5_ (Table 2); 44 studies were identified but only four assessed both post-bronchodilator airflow obstruction and quantified exposure to PM_2.5_.^31^–^34^ One of these studies was the cross-sectional analysis of the baseline Malawi study discussed above.^34^ A study from India showed a low prevalence of COPD and no association with biomass smoke exposure,^33^ and in a study from Tanzania with a substantial prevalence of COPD, the authors were not able to show an association because 99.5% of the study population was exposed to biomass smoke.^32^ In the study from Peru that did report an association between exposure to biomass PM_2.5_ and COPD, there was likely confounding with tobacco smoking, high altitude and age.^31^ Thus, recent studies in adults have not been particularly useful in shedding light on whether there is a mechanistic relationship between exposure to HAP and the development of COPD independent of other poverty-associated risk factors.

**Table 2:** Original research papers of HAP and COPD published since 2015 that assessed both post-bronchodilator airflow obstruction and quantified exposure to PM_2.5_

Study	Year	Study design	Population	Sample size	Definition of HAP exposure	COPD diagnosis	Post-BD FEV_1_
Brakema^31^	2019	Observational study	Highland (~2050 m above sea level) and a lowland (~750 m above sea level) setting in rural Kyrgyzstan	392 participants: 199 highlanders and 193 lowlanders	Indoor particulate matter was measured with an aerodynamic diameter <2.5 μm PM_2.5_	COPD was more prevalent among highlanders (36.7% vs. 10.4%; *P* < 0.001). Their average PM_2.5_ exposure was also higher (290.0 vs. 72.0 μg m 3; *P* < 0.001). In addition to high PM_2.5_ exposure (OR 3.174, 95% CI 1.061–9.493), the altitude setting (OR 3.406, 95% CI 1.483–7.825), pack-years of smoking (OR 1.037, 95% CI 1.005–1.070) and age (OR 1.058, 95% CI 1.037–1.079) also contributed to a higher COPD prevalence among highlanders	FEV_1_ and FVC before and after bronchodilation with 400 lg salbutamol using a spacer. COPD was defined as having a post-BD FEV_1_/FVC ratio <70% GOLD FEV_1_/FVC < 0.70
Magitta^32^	2018	Cross-sectional survey	Adults aged ≥35 years in rural Tanzania	869 participants	CO levels indoors and outdoors measured	Spirometry was performed and COPD diagnosed based on post- BD FEV_1_/FVC < 70%	Insufficient power to study the risk of biomass fuel smoke exposure for COPD as 99.5% of the total population was skewed towards the use of biomass fuel
Mahesh^33^	2018	Randomised observational study	Adults aged ≥30 years rural areas of Mysore District, in south India	8,457 patients with 1,085 tested for lung function; follow-up of 869 patients 5 years later	Indoor levels of CO and sulphur dioxide and nitric oxide during cooking and 3 hours thereafter were measured in 50 randomly chosen participant households	Evaluation of lung function and COPD by spirometry	COPD was defined according to the GOLD spirometry guidelines as a post-BD ratio of FEV1/FVC < 0.7
Nightingale^34^	2019	Cross-sectional (participants of RCT)	Adults (mean age 43.8 years) in rural Malawi	1,481	Personal exposure to PM_2.5_ and CO were measured continuously for 48 h using the Indoor Air Pollution (IAP) 5000 Series Monitor (Aprovecho Research Center, Cottage Grove, OR, USA)	Pre- and post-BD spirometry	No significant association between air pollution exposure and an increased risk of spirometric abnormalities

HAP=household air pollution; COPD=chronic obstructive pulmonary disease; PM_2.5_=particulate matter 2.5 microns or smaller in size; BD=bronchodilator; FEV_1_=forced expiratory volume in 1 sec; OR=odds ratio; CI=confidence interval; FVC = forced vital capacity; GOLD = Global Initiative for Chronic Obstructive Lung Disease; CO = carbon monoxide.

Looking earlier in life to the early origins of COPD, there are several studies of HAP exposure and lung function in children which provide evidence of impaired lung function in HAP-exposed children.^15^–^17^,^35^ Results from the CRECER Study in Guatemala (a follow-up to the RESPIRE Trial) suggested that reduced exposure to HAP through the use of a chimney stove intervention during pregnancy and infancy could improve lung function as measured by spirometry later in childhood.^16^ In the Ghana Randomized Air Pollution and Health Study (GRAPHS), 48-h personal monitoring of CO exposures was done four times over the course of pregnancy in a cluster-randomised intervention trial of cleaner-burning cookstoves, and lung function parameters, including passive respiratory system compliance with the single-occlusion technique, were measured in the infant offspring of the monitored mothers at 1 month of age. The primary finding was that maternal CO exposure during gestation was associated with lower infant lung function.^17^ In a sub-study of the Cooking and Pneumonia Study (CAPS), a trial of cleaner-burning biomass-fuelled cookstoves in Malawi, children from intervention households had lower carboxyhaemoglobin and higher forced vital capacity (FVC) than controls.^15^ While these studies have limitations similar to those discussed in the COPD literature, especially with regard to the snapshot nature of HAP exposure measurement, their results suggest that exposure to HAP in early life is a risk factor for poorer lung function, possibly COPD, later in life.

## IS THE PHENOTYPE OF FIXED AIRFLOW OBSTRUCTION ASSOCIATED WITH HAP SIMILAR OR DIFFERENT FROM TOBACCO-SMOKE COPD?

There is a school of thought according to which COPD should be redefined according to the underlying aetiology. Such an approach would be valuable in directing attention to the non-smoking-related types of COPD about which little is known (particularly in relation to morbidity, mortality and treatment) compared to tobacco smoking-related COPD. In contrast to some of the other types of COPD, COPD related to HAP exposure seems likely to have more in common with tobacco smoking-related COPD since tobacco is itself biomass. In fact, at least two studies have reported that the survival of spirometry-diagnosed biomass-related COPD in non-smokers is similar to that of tobacco-related COPD.^36^–^38^

However, as there are clear differences between the two (see Table 3 for summary), it would be wrong to extrapolate fully from tobacco smoking-related to HAP-related COPD. An additional complexity is that in settings where HAP is common, so are other drivers of COPD, including sub-optimal lung development in early life, tobacco smoking, infections (particularly TB) and poorly treated asthma. Pure HAP-related COPD may therefore be uncommon and is more likely to be observed as part of what might be termed a mixed aetiology COPD phenotype.

**Table 3:** Comparison of smoking- vs. HAP-related COPD^47^,^58^,^59^

Feature	Smoking-related	HAP-related
Epidemiology	Main cause worldwide	Mostly in LMICs and rural areas
Sex	Still predominates in men but increasing in women	Women (exposed domestically)
Context	Passive smoking relevant in early life, but active smoking is major driver from adolescence onwards	Typically women who never smoked cigarettes but had lifelong exposure to biomass smoke in a rural area
Pathology (macroscopic)	Emphysema, chronic bronchitis	More chronic bronchitis, less emphysema, anthracosis
Pathology (microscopic)	Emphysema, goblet cell hyperplasia	Fibrosis of small airways, intimal thickening of pulmonary arterioles, anthracosis
Symptoms	Breathlessness dominant, airway-related symptoms (cough, phlegm, wheezing) also common	Airway-related symptoms dominate
Lung function	Airflow obstruction more severe; low DL_CO_ common; bronchial hyperresponsiveness less common	Airflow obstruction milder; low DL_CO_ uncommon and mild; bronchial hyperresponsiveness more common
CT scanning	Airway changes with variable amounts of emphysema	Airway changes with no significant emphysema
Prevention	Tobacco control and smoking cessation; challenging due to nicotine addiction	Exposure avoidance, which is challenging due to poverty
Treatment	Several clinical trials including inhaled drugs, LABA, LAMA, inhaled corticosteroids	Treated as COPD due to smoking but with no clinical trials

HAP = household air pollution; COPD = chronic obstructive pulmonary disease; LMIC = low- and middle-income countries; DL_CO_ = diffusing capacity for carbon monoxide; CT =computed tomography; LABA = long-acting beta-agonist; LAMA = long-acting muscarinic antagonist.

Another issue on the theme of lung function and HAP is that lung function abnormalities other than fixed airflow obstruction appear to be more common in LMICs (where HAP exposure is also common).^25^,^39^ Low FVC appears to be particularly common, although defining low FVC is highly problematic due to the limitations of the available lung function reference ranges.^40^–^42^ Even less is known about the phenomenon of low FVC (e.g., in relation to morbidity, mortality, treatment) compared to airflow obstruction in LMICs. What does seem clear, however, is that low FEV_1_ and low FVC are both associated with increased mortality across multiple populations, with some evidence from the PLATINO study from three Latin-American sites^43^,^44^ that FEV_1_ is a better predictor of overall and cardiovascular mortality than FVC. The high burden of impaired lung function in LMIC populations is therefore of great concern irrespective of causality.

## WHERE DO WE GO FROM HERE?

There is no doubt that there is substantial preventable morbidity and mortality associated with both HAP and COPD as these are currently measured and incorporated into the GBD study and WHO estimates. They are also both strongly associated with poverty, which is itself a major cause of preventable morbidity and mortality. Ideally, we would be able to disentangle ‘air quality poverty’ from other forms of poverty, but these are conundrums within conundrums that limit our ability to do so. If we are to accept these imperfections and uncertainties, where does that leave us and where do we go from here?

It certainly leaves us with a substantial burden of preventable morbidity and mortality in need of solutions, so we suggest that the way forward is to look for search for such solutions. We recommend that this search focuses on the development and evaluation of the clinical and cost-effectiveness of interventions to address ‘air quality poverty’ and other dimensions of poverty associated with COPD as these point us on the path to improved lung health and beneficial impacts. Of note, almost all trials of interventions to address HAP have, to date, concentrated on cooking stove/fuel interventions and these have not yielded the expected health benefits.^45^,^46^ It is therefore important that interventions that go beyond cooking-related HAP exposures, explore how we can achieve clean (clean enough) air and examine how other dimensions of poverty can be addressed at the same time are developed and formally evaluated.

Interventions that are known to benefit people both at risk of developing and with established COPD are out of reach for the majority living in LMICs, and these need to be made more widely available and affordable.^4^ It will be important that as interventions are developed and implemented, this is done with careful consideration of cost-benefits, as well as impacts on women, the environment and planetary health.

A large multi-country randomised controlled intervention trial of liquified petroleum gas (LPG) is ongoing and hopefully, when completed, will shed some light on the efficacy of this intervention to improve the health of both children and adults.^47^ Even if shown to be efficacious, it will remain to be seen whether LPG can be feasibly provided to the poorest and most rural populations in LMICs. However, clean air during adulthood could be too late if the main impact of pollutants occurs during prenatal and postnatal lung growth and development. If this is the case, adequate trials should include at least intermediate outcomes at birth and during growth, increasing considerably the complexity and cost.^48^

Finally, there is a long history of intervention implementation (e.g., ‘improved cookstoves’) for HAP at great cost despite minimal (or no) direct evidence of health benefits; it is especially important to avoid such wasteful approaches and concentrate on the generation of a robust evidence base to ensure appropriate use of limited resources in order to address HAP and other dimensions of poverty.

## CONCLUSION

There is substantial preventable morbidity and mortality associated with HAP, COPD and poverty, separately and together. Although it may not be possible to establish clear causal links between HAP and COPD, there is a clear urgency to reduce the avoidable burden of disease they inflict on the world’s poor.
